# The Baleful Star

**Published:** 1872-09

**Authors:** 


### THE BALEFUL STAR

Of September sheds its malignant rays over humanity, infusing the poison of hateful disease and sudden death on half the globe, especially in our latitudes, those of the United States, because, mainly, the weather has been hot for weeks, evaporating winter streams and fresh-water ponds, leaving exposed to the sun's rays their soft, wet, slimy bottoms, generating miasmatic influences, disease-engendering emanations, which enter into the circulation through the stomach and lungs, poison the blood, thickening it, making it congest in the small terminal vessels, laying the foundation at once of diarrhœa, dysentery, and every class of fevers, from the comparatively undangerous ague, to the malignant bilious, typhoid, and yellow jack.

One half of all these diseases could be prevented at one swoop by cleanliness and scientific draining. Look at New Orleans, during the Federal occupation of the war. One party seemed to think that the merciful One had forsaken them, the other that they were the special favorites of Heaven; both were equally wide of the mark; it simply was the secret of one intelligent mind, compelling clean streets, and what was equivalent to a constant drain.

Many a farm, with as rich a soil as the Delta of Egypt, cannot be sold at ten dollars an acre, because it is

A SICKLY HOLE; fever and ague reigns rampant; with a scientific draining, it would be worth a hundred dollars an acre, the very first crop paying the expense of drainage.

But it is too late to talk of draining, when whole families are shaking with ague, others growing as yellow as pumpkins, others again sinking under typhoid.

All the ailments named are bilious ailments, are the result of an inactive liver; there is so much blood dammed up in it, that it has no room to work, to free itself, no more than a man can use his elbows for deliverance when urged onwards to the door of a building on fire by the affrighted crowd around him. The physician understands what remedies are needed under the circumstances. The allopath is enthusiastic on his calomel; the water-cures slosh away with might and main, inside and out, top and bottom, with a free through ticket thrown in, while the infinitesimal folk give a mite a month, throwing the whole responsibilities of the case upon the broad shoulders of their

OLD STAND-BY,

Doctor Nature; and I tell you what, that same old Doctor don't always come out second best; he has a way of his own, and waddles along, as in the celebrated race between Messrs Turtle and Hare, a long time ago, in such a way as to come out, half a neck ahead, colors flying.

It is very true that a pour bath of ice-water or even water just from the spring, will break up the chill, and cure the disease in many cases, but the remedy is little less than terrible; few would submit to a second operation, and some have died in its progress. That mercury is the most infallible agency known to man, is an accepted fact; but many have a prejudice against its use; meanwhile, we will leave it to the tincture of time, and the little pellets, to cure all who are suffering now, it being a main province of this journal to prevent these diseases.

Places, neighborhoods, now under the bane of miasmatic emanations, should be drained before another season, ploughed over and put under cultivation, or filled up, after a winter and half summer exposure.

But a few practical facts in reference to the nature of miasm and its laws will bear an annual repetition, until the people can be made to apprehend them, and act intelligently in reference to them.

Miasm, the one great cause of prevalent epidemics, from the simplest fever or diarrhœa to malignant Asiatic cholera, is most pernicious, almost exclusively so, for the hour including sunrise and sunset, there being a dampness, rawness, and heaviness in the atmosphere at such times not found at midday or midnight, leading to the practice, in intelligent families where these autumnal diseases prevail, of having a blazing fire on the hearth at sunrise and sunset, in the family room, where the family should be gathered of course; the cheery fire antagonizes the influences named, changes the physical condition and constitution of the atmosphere. But there is an additional precaution, and both together have amounted to a total exemption from the diseases in whole families, simply by arranging to take breakfast before going outside the door in the morning, getting back into the house a little before sundown, and sitting down to a hot supper as soon as reaching home, at least something hot enough to wake up the circulation of the body, and thus repress the influences of disease, until the digestion of the sufferer commences to impart nutriment and strength to the system. It is peculiarly appropriate in the morning, where fever and ague and similar diseases prevail, to take something hot into the stomach, a cup of coffee or tea, or chocolate or broma ; these are best, especially if a bit of cracker or bread is added. In many cases, one or two oranges or apples, or dry cracker or lemon are sufficient, because they excite the secretions of the stomach, warm it up, wake it into action, and thus prevent the absorption of poisonous gases, giving in their stead healthful nutriment to the blood, and not the baleful miasm.

